# Diagnostic validation of the Chinese version of the five-part questionnaire for screening joint hypermobility in young adults

**DOI:** 10.1038/s41598-026-45970-8

**Published:** 2026-03-26

**Authors:** Yu Wang, Xin Li, Yiduo Wang

**Affiliations:** 1 School of Sports Medicine and Health, Chengdu Sport University, 1942 North Huanhu Road, Chengdu, China; 2 Department of Surgery and Cancer, Faculty of Medicine, Imperial College London, 86 Wood Lane, London, W12 0BZ UK

**Keywords:** Joint Instability, Beighton score, Five-part questionnaire (5PQ) for joint hypermobility, Reliability, Validity, Chinese, Diseases, Health care, Medical research

## Abstract

Joint hypermobility shows considerable variability across populations, yet validated screening tools for Mandarin-speaking cohorts are lacking. The Five-Part Questionnaire (5PQ) is widely used internationally, but its properties have not been evaluated in Chinese populations. This study assessed the validity and reliability of the Chinese version of the 5PQ (5PQ-CN) in a cohort of young Chinese university students. Participants completed the 5PQ-CN and underwent clinician-administered Beighton Score (BS) assessment, using the internationally recommended cut off of ≥ 5 to identify generalised joint hypermobility (GJH). Diagnostic validity was examined using sensitivity, specificity, predictive values, accuracy, and the area under the receiver operating characteristic curve (AUC). Test-retest reliability was evaluated using Cohen’s kappa (κ) and intraclass correlation coefficients (ICC). A total of 1,910 participants were recruited; 615 were included in the validity analysis and 325 in the reliability subgroup. The 5PQ-CN showed 78.6% sensitivity, 65.8% specificity, 72.2% accuracy, and an AUC of 0.722. Test-retest reliability demonstrated substantial agreement (κ = 0.703) and moderate consistency for the total score (ICC = 0.707). The 5PQ-CN is a valid and reliable self-report instrument for screening GJH in young Chinese adults within a university setting. Its high feasibility and sensitivity make it a useful complement to clinician-based assessments, particularly in large-scale or resource-limited epidemiological settings.

## Introduction

 Joint hypermobility is defined as a condition in which one or more joints move beyond the normal physiological range of motion and may be associated with hereditary connective tissue disorders^1^. Asymptomatic hypermobility, also referred to as generalised joint hypermobility (GJH), is reported between 2% and 57% of the population^2,3^. The wide variation in prevalence reporting can be affected by different assessment methods, measurement thresholds, and population differences^4^, which highlights the importance of standardization of screening tools. Existing evidence suggests asymptomatic hypermobility is more common in young women cohort^5,6^, as well as certain racial groups such as Africans^7^ and Arabic^8^. However, it remains unclear whether existing screening tools are equally valid in larger racial cohorts such as Asians, necessitating further validation.

Asymptomatic hypermobility can be beneficial in activities requiring high flexibility, including music performance^9^, gymnastics^10^, and dance^11^. Although asymptomatic hypermobility is not pathological, distinguishing it is essential for identifying the other hypermobile subgroup who presents with symptomatic hypermobility, classified as either hypermobility spectrum disorders (HSD)^12^ or hypermobile Ehlers-Danlos syndrome (hEDS)^13^. People with symptomatic hypermobility typically present with multisystemic clinical manifestations alongside joint hypermobility, including but not limited to joint instability^14^, chronic pain^15^, central fatigue^16^, postural tachycardia syndrome^17^, gastrointestinal symptoms and nutritional issues^18^, anxiety disorders^19^, abnormal skin elasticity and scarring^20^, urinary complications^21^, fall and imbalance^22^. These cases are associated with substantial medical (e.g., $32,800 per patient in the U.S.) and social costs^23^, However, early identification depends heavily on reliable screening tools. Given the multisystemic nature of symptomatic hypermobility, early identification of at-risk individuals requires reliable epidemiological screening tools, such as the Beighton score (BS) and the Five-Part Questionnaire (5PQ).

The BS is a clinician-administered 9-point scale^24^, assessing joint mobility by using goniometric measures^25^. In contrast, the 5PQ is a self-reported assessment tool comprising five questions related to generalised joint hypermobility^26^. The 5PQ has been validated in several populations^27–29^. It is known joint hypomobility is more common in Asian cohort, whereas several studies have adopted a BS cut-off of ≥ 4 to define asymptomatic hypermobility in young Asian adults^30,31^. Their threshold may artificially increase false positive rates in groups^32^, and such may also confuse the characteristics of asymptomatic young adults with those of children who also adopted a BS cut-off of ≥ 4 based on the 2017 international classification criteria. Although the criteria recommends a cut-off of ≥ 5 for adults^13^, there is a lack of studies evaluating validity and reliability properties of BS and 5PQ at this threshold, limiting evidence-based recommendations in order to determine the definitive assessment on classifying asymptomatic hypermobility for clinical use. Although Portuguese and Swedish versions of the 5PQ have been validated, psychometric data indicate that cross-cultural adaptation may impact comprehensibility and sensitivity and its measurement equivalence cannot be assumed to exist and needs to be further verified^28,29^. Therefore, the Mandarin version to fit Chinese great population need to be validated.

To address these gaps, the present study aims to apply the internationally recommended cut-off (BS ≥ 5) as a reference standard to evaluate the validity and reliability of the Chinese version of the 5PQ (5PQ-CN) against with the established BS for assessing asymptomatic hypermobility in adults, thereby reducing epidemiological inconsistencies caused by differences in tools in prior studies. 5PQ-CN may serve as a useful screening instrument for the great Mandarin-speaking Asian population and support further evidence-based recommendation in primary healthcare, population research, sports medicine screening, and other scenarios.

## Methods

### Study design

This prospective diagnostic validation study consisted of two sessions: an initial visit and a follow-up testing session conducted at a seven-day interval. This duration was chosen to minimize memory effects while ensuring clinical stability. Given that GJH is a structural physical trait primarily determined by age, sex, and ethnicity, it is considered stable over such a short period in the absence of acute injury or intervention. A subgroup of participants was assigned to the test-retest reliability analysis and therefore attended both sessions, while all other participants attended only the main session.

**Fig. 1:**
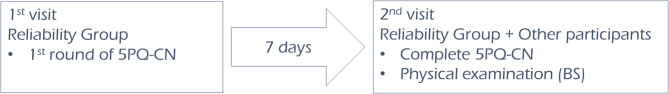
Testing procedure. 5PQ-CN: Chinese version of five-part questionnaire, BS: Beighton Score.

At the initial visit, participants in the reliability subgroup completed the 5PQ-CN and a demographic questionnaire. Seven days later, during the main testing session, all participants completed the 5PQ-CN. Participants who had not attended the initial visit completed the demographic questionnaire at this time. After questionnaire completion, all participants underwent a physical assessment for GJH using the Beighton Score (BS). The reliability subgroup completed this physical assessment only once, after their second administration of the 5PQ-CN. To ensure consistency, both measurements were administered by the same researcher using standardised instructions and identical assessment protocols. All tests were conducted in a controlled environment to minimize external variables that could influence joint hypermobility or participant responses Fig. 1.

### Participants

Participants were recruited in November 2021 from two universities in Chengdu, China, using convenience cluster sampling. Lecturers from relevant disciplines, including rhythmic gymnastics, sports dance, basketball, football, tennis, sport science, and sport rehabilitation science, were contacted to invite students to participate.

The inclusion criteria were:^1^ good general health;^2^ provision of informed consent;^3^ no acute injuries or chronic conditions that could interfere with the Beighton Score (BS) assessment or the 5PQ-CN; and^4^ age 18 years or older. The exclusion criteria were:^1^ inability to undergo joint examinations;^2^ other physical disabilities that could interfere with the assessment.

A total of 615 participants were enrolled. Informed consent was obtained at the start of each testing session. The study received ethical approval from the Research Ethics Committee of Chengdu Sport University (Approval No. [2021] 46) and was conducted in accordance with the Declaration of Helsinki.

A priori sample size estimation was performed using G Power version 3.1.9.7. Based on prevalence, sensitivity, and specificity values reported by a previous study with alpha set at 0.05 and power set at 0.90^28^, the minimum required sample size was 227. To account for possible participant drop out, the target sample size was increased to 454.

### Assessment instruments

The 5PQ-CN was used as the index test. The original English version of the Five-Part Questionnaire, developed by Hakim and Grahame^26^, is a five-item self-report measure that asks whether participants can perform specific joint movements. Each “yes” response receives one point, and a total score of 2 or more indicates GJH in adults^26^. The translation process was conducted as a formal cross-cultural adaptation. Specifically, the 5PQ-CN was translated into Chinese by a certified translator and back-translated into English by an independent translator. The content validity of the 5PQ-CN was then reviewed for accuracy and conceptual equivalence by an expert committee which compromises physiotherapists, methodologists and linguistic experts. Subsequently, pre-testing was performed with six stakeholders to ensure that the Chinese phrasing was easily understood and culturally appropriate for the target population. The original English items and the Chinese translations are shown in Table 1. Clarifications were added to Item 4 by specifying the patella and to Item 5 by adapting the wording to “Do you consider your joints to be hypermobile (excessively flexible).”

**Table 1:** The five part questionnaire and its Chinese translation.

Items	5PQ	5PQ-CN
1	Can you now [or could you ever] place your hands flat on the floor without bending knees?	您现在或曾经是否可以在不弯曲膝盖的情况下将双手手掌展开、向下平放在地面上༟
2	Can you now [or could you ever] bend your thumb to touch your forearm?	您现在或曾经是否可以将您的大拇指弯曲到触碰您手臂的程度༟
3	As a child, did you amuse your friends by contorting your body into strange shapes or could you do the splits?	当您还是孩子的时候, 您是否曾经将您的身体扭曲成奇怪的形状来逗您的朋友开心༟或者您可以做劈腿动作吗༟
4	As a child or teenager, did your kneecap or shoulder dislocate on more than one occasion?	在您还是孩子或青少年的时候, 你的肩膀或者髌骨(膝盖)是否曾经不止一次地脱臼༟
5	Do you consider yourself “double-jointed”?	您觉得您自己的关节过度灵活吗༟

5PQ, Five-Part Questionnaire; 5PQ-CN, Chinese version of the Five-Part Questionnaire. The original English version was developed by Hakim and Grahame^26^.

**Fig. 2:**
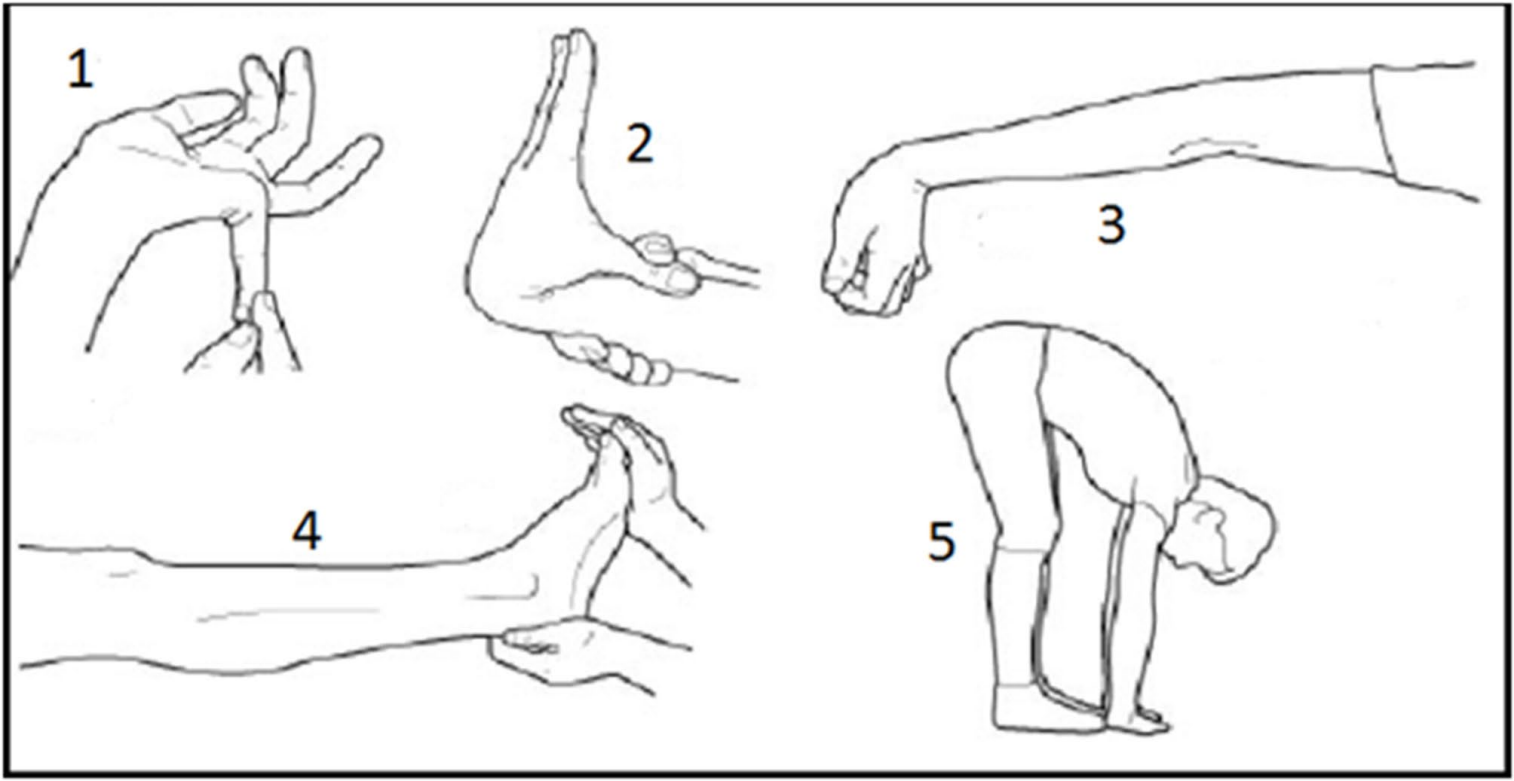
Beighton Score assessment illustration. Positive criteria include:^1^ joint angle of at least 90 degrees;^2^ thumb touching the forearm;^3^ elbow hyperextension of at least 10 degrees;^4^ knee hyperextension of at least 10 degrees; and^5^ placing both palms flat on the floor without bending the knees. Items 1 to 4 are assessed bilaterally. Illustration adapted from Physiopedia, Hypermobility Syndrome^34^.

The BS is used as the reference standard for GJH^24,32^. This assessment includes nine measures of upper limb, lower limb, and spinal flexibility (Fig. 2). Each item is scored as positive or negative, producing a total score from 0 to 9. Following the 2017 international classification^13^, a score of 5 or more was used to identify GJH in adults between 18 years old and 50 years old. Although previous studies have questioned the reliability of the BS^24,32,33^, it remains the most widely accepted clinical measure. All assessments were conducted by trained researchers who were blinded to questionnaire responses.

### Data processing and statistical analysis

Data were processed using Microsoft Excel (2019) and IBM SPSS Statistics version 25. Continuous variables are presented as mean ± standard deviation, and categorical variables as frequency and percentage with a 95% confidence interval. Statistical significance was defined as p less than 0.05.

Diagnostic performance of the 5PQ-CN relative to the BS was examined using accuracy, sensitivity, specificity, positive predictive value, negative predictive value, and receiver operating characteristic analysis. The area under the curve was interpreted as excellent (0.90 to 1.00), considerable (0.80 to 0.89), fair (0.70 to 0.79), poor (0.60 to 0.69), or fail (0.50 to 0.59)^35^.

For item level analysis, chi square tests were used to compare the frequency of positive responses across BS based groups, and odds ratios with 95% confidence intervals were calculated. Test-retest reliability within the reliability subgroup was evaluated using Cohen kappa for dichotomous items and the intraclass correlation coefficient (two-way mixed model, absolute agreement; ICC^1,3^ for total scores^36,37^. McNemar test was used to detect changes in paired item responses.

## Results

### Participants and analysis flow

**Fig. 3:**
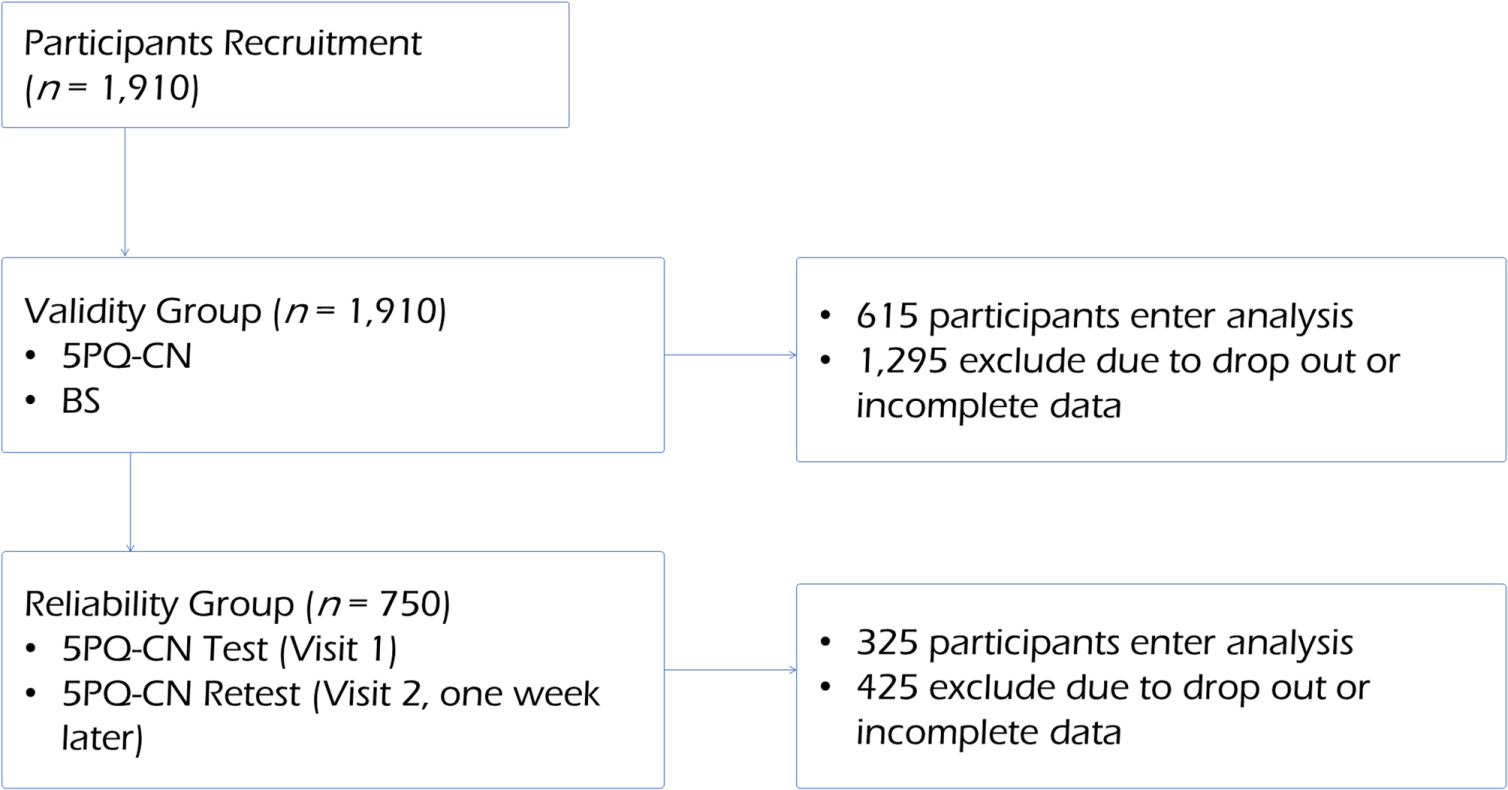
Participant flow. 5PQ-CN: Chinese version of the Five-Part Questionnaire, BS: Beighton Score.

Figure 3 illustrates the participant flow. Initially, 1,910 participants were recruited. A subgroup of 750 participants was selected for the test-retest reliability analysis. Of these, 325 participants completed both rounds of the 5PQ-CN and were included in the reliability analysis, while 425 participants were excluded due to missing data or absence from the second session. For the validity analysis, all 1,910 participants were screened. After excluding those who did not complete the physical assessment or had missing data, 615 participants were included in the final validity sample.

The demographic data for the validity and reliability groups are presented in Table 2. The validity group consisted of 615 participants (age: 19.6 ± 1.38 years; height: 1.71 ± 0.09 m; weight: 61.06 ± 11.94 kg; BMI: 20.86 ± 3 kg·m⁻²), including 286 (46.5%) males and 329 (53.5%) females. The reliability group consisted of 325 participants (age: 19.84 ± 1.46 years; height: 1.71 ± 0.09 m; weight: 62.09 ± 11.9 kg; BMI: 20.99 ± 2.75 kg·m⁻²), including 139 (42.8%) males and 186 (57.2%) females.

**Table 2:** Demographics of participants.

Variables	Validity Group			Reliability Group		
	Male	Female	Total	Male	Female	Total
Number	286 (46.5%)	329 (53.5%)	615 (100%)	139 (42.8%)	186 (57.2%)	325 (100%)
Age (years)	19.62 ± 1.35	19.59 ± 1.39	19.6 ± 1.38	19.99 ± 1.42	19.73 ± 1.49	19.84 ± 1.46
Height (m)	1.77 ± 0.06	1.65 ± 0.07	1.71 ± 0.09	1.78 ± 0.07	1.67 ± 0.07	1.71 ± 0.09
Weight (kg)	68.36 ± 11.04	54.7 ± 8.59	61.06 ± 11.94	70.77 ± 9.36	55.61 ± 9.15	62.09 ± 11.9
BMI (kg·m^− 2^)	21.84 ± 3.09	20.01 ± 2.64	20.86 ± 3	22.39 ± 2.4	19.94 ± 2.52	20.99 ± 2.75

BMI: body mass index.

### GJH prevalence

The prevalence of GJH as identified by the BS and the 5PQ-CN is shown in Table 3. Based on a BS cutoff of ≥ 5, 308 participants (50.1%) were classified as GJH-positive. Using a 5PQ-CN cutoff of ≥ 2, 347 participants (56.4%) were classified as positive for GJH. A significant difference was observed in the GJH diagnosis between the two methods (*χ²* = 10.148, *p* = .001).

**Table 3:** GJH prevalence accessed by both tools.

GJH status	BS	5PQ-CN
Positive	308 (50.1%)	347 (56.4%)
Negative	307 (49.9%)	268 (43.6%)
Total	615 (100%)	615 (100%)

GJH: generalized joint hypermobility, BS: Beighton score, 5PQ-CN: Chinese version of five-part questionnaire.

### Validity

The diagnostic performance of the 5PQ-CN relative to the BS is summarized in Table 4. Among the 347 participants with a positive 5PQ-CN result, 242 were true positives and 105 were false positives, yielding a PPV of 69.74% (95% CI: 64.89–74.59). Among the 268 participants with a negative 5PQ-CN result, 202 were true negatives and 66 were false negatives, yielding a NPV of 75.37% (95% CI: 70.20-80.54). The overall accuracy of the 5PQ-CN was 72.19% (95% CI: 68.64–75.74), with a sensitivity of 78.57% (95% CI: 74.00-83.14) and a specificity of 65.80% (95% CI: 60.48–71.12). The AUC of ROC was 0.722, indicating moderate diagnostic accuracy for the 5PQ-CN.

**Table 4:** GJH Diagnosis by 5PQ-CN.

Table 4. GJH Diagnosis by 5PQ-CN			
GJH status	TRUE	FALSE	Sum
Positive	242 (39.3%)	105 (17.1%)	347 (56.4%)
Negative	202 (32.8%)	66 (10.7%)	268 (43.6%)
Total	444 (72.2%)	171 (27.8%)	615 (100%)

GJH: generalized joint hypermobility.

Table 5 presents the item-specific distribution of 5PQ-CN responses stratified by the BS diagnosis of GJH. *χ²* tests revealed a significant difference in the proportion of positive responses between GJH-positive and GJH-negative groups for items 1, 2, 3, and 5 (*p* < .001), but not for item 4 (*p* = .547). The highest *OR* was observed for item 2 (*OR* = 8.09, 95% *CI*: 4.86–13.45), followed by item 1 (*OR* = 5.24, 95% *CI*: 3.53–7.78) and item 3 (*OR* = 5.01, 95% *CI*: 3.55–7.05). Item 5 had the lowest *OR* (*OR* = 2.77, 95% *CI*: 1.74–4.41) among the items showing significant differences.

**Table 5:** 5PQ-CN item-specific distribution grouped by.

Items	GJH Positive		GJH Negative		OR	95% CI	
	yes	no	yes	no			
1^*^	266 (43.3%)	42 (6.8%)	168 (27.3%)	139 (22.6%)	5.24	3.53	7.78
2^*^	111 (18%)	197 (32%)	20 (3.3%)	287 (46.7%)	8.09	4.86	13.46
3^*^	217 (35.3%)	91 (14.8%)	99 (16.1%)	208 (33.8%)	5.01	3.56	7.06
4	25 (4.1%)	283 (46%)	21 (3.4%)	286 (46.5%)	1.20	0.66	2.20
5^*^	69 (11.2%)	239 (38.9%)	29 (4.7%)	278 (45.2%)	2.77	1.74	4.41

*: *p* < .001 in χ^2^, GJH: generalized joint hypermobility, OR: odds ratio, CI: confidence interval.

### Test-retest reliability

The consistency of the 5PQ-CN diagnoses between the two rounds of administration is shown in Table 6. In the first round, 179 participants (55.1%) were classified as GJH-positive and 146 (44.9%) as GJH-negative. In the second round, 171 (52.6%) were positive and 154 (47.4%) were negative. No significant difference was observed in the distribution of diagnoses or the mean cumulative scores between rounds (*p* ≥ .05). Cohen’s κ for the dichotomous diagnosis was 0.703, indicating substantial reliability. The ICC for the total score was 0.707 (95% CI: 0.649-0.758; single-measurement, absolute agreement, two-way mixed-effects model), indicating moderate reliability.

**Table 6:** 5PQ-CN test-retest reliability.

Variables	1st round	2nd round
Positive	179 (55.1%)	171 (52.6%)
Negative	146 (44.9%)	154 (47.4%)
Total	325 (100%)	325 (100%)
Mean accumulative score	1.65 ± 1.18	1.61 ± 1.18

Item-specific reliability results are shown in Table 7. Item 3 had the highest proportion of response shifts, with 63 participants (19.4%) altering their response between rounds. This was followed by item 2 (48 participants, 14.8%) and item 1 (46 participants, 14.2%). Item 4 had the lowest number of response changes (18 participants, 5.5%). McNemar’s test indicated a significant difference in the response distribution for items 2 and 3 (*p* < .001). Cohen’s κ values for individual items ranged from 0.442 to 0.664, indicating moderate to substantial reliability.

**Table 7:** Item-specific distribution of 5PQ-CN in both round of test.

Items	1st Round		2nd Round		Changed responses	kappa
	yes	no	yes	no		
1	231 (71.1%)	94 (28.9%)	223 (68.6%)	102 (31.4%)	46 (14.2%)	0.664^^^
2^*^	41 (14.5%)	278 (85.5%)	79 (24.3%)	246 (75.7%)	48 (14.8%)	0.535^^^
3^*^	180 (55.4%)	145 (44.6%)	151 (46.5%)	174 (53.5%)	63 (19.4%)	0.615^^^
4	27 (8.3%)	298 (91.7%)	21 (6.5%)	304 (93.5%)	18 (5.5%)	0.596^^^
5	52 (16%)	273 (84%)	50 (15.4%)	275 (84.6%)	48 (14.8%)	0.442^^^

*: *p* < .001 in McNemar’s test, ^: *p* < .001 for kappa.

## Discussion

This study evaluated the validity and reliability of the 5PQ-CN for screening asymptomatic joint hypermobility (GJH) in young Chinese adults, using the BS with the internationally recommended cut off of ≥ 5 as the reference standard^13^. By applying the adult threshold proposed in the 2017 international classification^13^, this study addresses concerns regarding inconsistent BS cutoffs in Asian cohorts^30,31^ and contributes to the standardisation of hypermobility screening practices. To our knowledge, this is the first validation of the 5PQ in a Chinese population, which is important given ethnic variation in joint hypermobility^2^ and the absence of psychometric evidence supporting the use of Western developed tools in Asian populations^28,29^.

The prevalence of GJH in this sample was higher than that commonly reported in Western cohorts (10–30%)^24,28^. This is consistent with the characteristics of the sample, which included young adults, a group known to have greater joint hypermobility, and a higher proportion of women, who are more likely to exhibit GJH^5^. Ethnicity may also have influenced prevalence, as previous studies indicate that hypermobility is more frequent in Asian populations than in Caucasian groups^2,12^. In addition, many participants had long term training in activities requiring high flexibility (e.g., gymnastics and dance)^38^, which may increase joint hypermobility. In cohorts with sport-related backgrounds, a certain degree of joint hypermobility is frequently a selected trait that presents a competitive advantage in specific disciplines. Consequently, the observed high prevalence shows the clinical utility of the 5PQ-CN as an efficient first-stage screening tool. Identifying GJH-positive individuals within these high-prevalence groups allows clinicians to proactively monitor for multisystemic ‘red flags’ and implement targeted injury-prevention strategies.

The diagnostic performance of the 5PQ-CN demonstrated moderate accuracy relative to the BS. Sensitivity (78.57%) was higher than that reported for the Brazilian Portuguese^29^ and the Swedish version^28^, while specificity and AUC were slightly lower. These differences may reflect cross-cultural variation, differences in sample composition, and the use of different BS thresholds across studies. Notably, this study used BS ≥ 5, consistent with international criteria^13^, while several earlier validations used BS ≥ 4^30,31^. By adopting BS ≥ 5, this study helps reduce epidemiological variation arising from inconsistent reference standards.

Item-level analysis showed that all items except item 4 distinguished between GJH-positive and GJH-negative participants. The low positive rate for item 4 aligns with previous work^25^, which attributed this pattern to the non-clinical nature of the sample, unlike the original 5PQ study^26^. Items 1, 2, and 3 had strong associations with BS-defined GJH, whereas item 5 had a comparatively lower odds ratio. This may reflect normalisation of flexibility among participants with sports training backgrounds, potentially reducing subjective awareness of hypermobility. In contrast, the Swedish cohort showed higher OR values for item 5^28^.

Comparisons between 5PQ-CN responses and BS assessments showed discrepancies attributable to differences between self-report and clinician-assessed measures. For example, some participants appeared to classify trunk flexibility positively when touching the floor with fingertips rather than placing palms flat, as required by the BS. This discrepancy may be due to cultural or subjective interpretations of the movement. Such a tendency toward over-reporting suggests that the conceptual threshold for ‘normal’ versus ‘hypermobile’ flexibility varies among laypersons. This scenario underscores the importance of incorporating standardised visual illustrations to minimise interpretative bias in self-administered tools. Similarly, incorrect execution of the thumb-to-forearm manoeuvre contributed to inconsistencies. These findings, also noted in previous psychometric work^39^, highlight limitations of self-administered tools and suggest that adding diagrams or clearer instructions may improve response accuracy.

Test-retest analysis demonstrated moderate reliability of the 5PQ-CN, with Cohen’s κ = 0.703 and ICC = 0.707. Item-level κ values ranged from 0.442 to 0.664, indicating moderate to substantial agreement, though slightly lower than those reported in the Brazilian Portuguese^29^ and Swedish versions^28^. Variability may reflect differences in training background or subjective interpretation.

Although the BS is well established, its requirement for trained examiners and sequential assessment limits feasibility for large-scale epidemiological use. In contrast, the self-administered 5PQ-CN can be distributed simultaneously to large groups, substantially reducing resource demands. The high sensitivity of the 5PQ-CN supports its utility as a first-stage screening tool for identifying individuals who may require further clinical assessment for symptomatic hypermobility, including HSD and hEDS. The NPV of 75.37% suggests that the 5PQ-CN functions effectively as a ‘rule-out’ assessment, which may allow clinicians to bypass more time-consuming physical examinations with reasonable confidence in low-risk or primary care settings. In contrast, the PPV of 69.74% indicates approximately 30% of those screening positive may be false positive. Therefore, a positive 5PQ-CN result may be interpreted as a trigger for the next stage physical assessment rather than a definitive diagnosis. This approach containing initial screening followed by targeted physical evaluation could optimizes clinical workflow and resource allocation in great population areas. However, the moderate specificity suggests that false positives should be expected when estimating population prevalence. A large sample size and use of BS ≥ 5 may enhance the methodological robustness of the study.

This study has few limitations. The sample consisted entirely of young university students from a single region in south-western China, a few of whom have sport-related background, which limits generalisability to more diverse populations. The text-only questionnaire format may have contributed to misinterpretation of items, particularly those assessing wrist/thumb and trunk flexibility. Regarding data management, the study relied on the compromise of self-reported data against objective assessment, which might lack the precision of real-time judgement for discrepant cases. In addition, the sample comprised asymptomatic individuals and does not permit conclusions regarding symptomatic populations. Finally, although the translation and cultural adaptation of the 5PQ were conducted by a multidisciplinary committee comprising clinical experts, methodologists, and patient stakeholders, this study did not strictly adhere to every procedural step outlined by the Consensus-based Standards for the selection of health Measurement Instruments (COSMIN) guidelines^40^.

Future research should validate the 5PQ-CN in wider age groups and regions, including the incorporation of age-dependent BS cutoffs. Adding visual illustrations may improve item comprehension. Additionally, further work should assess the questionnaire’s performance in clinical cohorts and its predictive validity for musculoskeletal or systemic outcomes. Such studies should strictly adhere to established methodological guidelines, such as COSMIN, to ensure the highest level of rigour in the validation process across diverse Mandarin-speaking populations.

## Conclusion

The 5PQ-CN demonstrated moderate diagnostic accuracy and moderate test-retest reliability when evaluated against the BS using the internationally recommended cut off of ≥ 5. As a self-administered instrument, it offers a practical and resource-efficient option for large-scale screening of GJH in young Chinese adults, complementing but not replacing clinician-administered assessments. The findings support the potential use of the 5PQ-CN as an initial screening tool in epidemiological research and population-based settings, where its high feasibility and sensitivity may facilitate early identification of individuals who could benefit from further evaluation. Wider application of the 5PQ-CN may help standardise hypermobility screening in Mandarin-speaking populations and contribute to improved understanding of GJH prevalence and related clinical pathways in China.

## Data Availability

The datasets generated and/or analysed during the current study are available from the corresponding author on reasonable request.

## Abbreviations

AUC: Area under the curve
HSD: Hypermobility spectrum disorders
hEDS: Hypermobile Ehlers-Danlos syndrome
BMI: Body mass index
BS: Beighton score
CI: Confidence interval
χ2: Chi-square
5PQ: Five-Part Questionnaire
5PQ-CN: Chinese version of the Five-Part Questionnaire
GJH: Generalised joint hypermobility
ICC: Intraclass correlation coefficient
κ: Cohen’s kappa
NPV: Negative predictive value
OR: Odds ratios
*p*: *P* value
PPV: Positive predictive value
ROC: Receiver operating characteristic
COSMIN: Consensus-based Standards for the selection of health Measurement Instruments
